# Fatigue Performance of ABS Specimens Obtained by Fused Filament Fabrication

**DOI:** 10.3390/ma11122521

**Published:** 2018-12-11

**Authors:** Miquel Domingo-Espin, J. Antonio Travieso-Rodriguez, Ramn Jerez-Mesa, Jordi Lluma-Fuentes

**Affiliations:** 1 Fundació Eurecat, Av. Universitat Autònoma, 23, 08290 Cerdanyola del Vallès, Spain; miquel.domingo@eurecat.org; 2 Mechanical Engineering Department, Escola d’Enginyeria de Barcelona Est, Universitat Politècnica de Catalunya, Avinguda d’Eduard Maristany, 10-14, 08019 Barcelona, Spain; 3 Engineering Department, Faculty of Sciences and Technology, Universitat de Vic-Universitat Central de Catalunya, C. Laura, 13, 08500 Vic, Spain; ramon.jerez@uvic.cat; 4 Materials Science and Metallurgical Engineering Department, Escola d’Enginyeria de Barcelona Est, Universitat Politècnica de Catalunya, Avinguda d’Eduard Maristany, 10-14, 08019 Barcelona, Spain; jordi.lluma@upc.edu

**Keywords:** parts design, additive manufacturing, fused filament fabrication, fatigue, Taguchi, ABS

## Abstract

In this paper, the fatigue response of fused filament fabrication (FFF) Acrylonitrile butadiene styrene (ABS) parts is studied. Different building parameters (layer height, nozzle diameter, infill density, and printing speed) were chosen to study their influence on the lifespan of cylindrical specimens according to a design of experiments (DOE) using the Taguchi methodology. The same DOE was applied on two different specimen sets using two different infill patterns—rectilinear and honeycomb. The results show that the infill density is the most important parameter for both of the studied patterns. The specimens manufactured with the honeycomb pattern show longer lifespans. The best parameter set associated to that infill was chosen for a second experimental phase, in which the specimens were tested under different maximum bending stresses so as to construct the Wöhler curve associated with this 3D printing configuration. The results of this study are useful to design and manufacture ABS end-use parts that are expected to work under oscillating periodic loads.

## 1. Introduction

Additive manufacturing (AM) technologies were, for years, considered only to manufacture prototypes, not end-use or functional objects. However, since the growth of the industry in the past years due to the improvement in technologies, the increasing quantity of materials, and the ease of access to the technologies, interest in manufactured functional parts has increased [1].

In order to manufacture a 3D object with AM, a virtual design is needed. Normally, the virtual design is done using computer aided design (CAD) software. After modeling the CAD file, the geometry is exported to an STL file, which describes the surface geometry of a three-dimensional object without any representation of color, texture, or other common model attributes. The STL file must be prepared before it is 3D printed, as it must be sliced. Slicing is dividing the 3D model into the horizontal layers that the printer will stack to form the part.

The first step before slicing is to orientate the part, which means how to place the part referred to the printer axis (X, Y, and Z). The orientation affects the surface roughness and/or dimensional accuracy [2,3,4,5,6,7,8,9,10], printing time [4,5,6], and part strength [7,10,11,12,13,14,15,16,17,18,19,20,21,22,23,24,25,26].

Slicing allows you to set several other printing parameters whose values affect the performance and characteristics of the part. Their values are critical in FFF technologies, as they affect the surface finish [27,28,29,30,31,32,33,34,35], cost [28,30,31,32,34,36], and mechanical performance [12,35,37,38,39,40,41].

The mechanical properties of FFF manufactured parts are difficult to predict, mainly because the parts present anisotropic mechanical behavior [16,23,25,42,43,44], and the printing parameters affect their mechanical response, the most studied being the layer height [19,35,39,41,45,46,47,48,49,50], infill orientation [13,18,21,23,35,44,48,50,51,52,53,54], infill pattern [13,24,41,46,53,55,56,57], infill density [13,35,38,41,44,45,46,49,57], wall thickness [22,23,45], and nozzle diameter [41,44].

Not many fatigue studies on AM manufactured parts have been reported. Most of them focus on metallic parts, as their applications require knowing the number of cycles to failure [15,26,58,59,60]. The combination of platform heating and peak-hardening on the selective laser melting (SLM) parts of AlSi10Mg increased the fatigue resistance and neutralized the differences in the fatigue life for different building orientations [15]. Also, the fatigue life of Ti-6Al-4V alloys fabricated by electron beam melting (EBM) and laser beam melting (LBM) was investigated. The results indicated that the LBM Ti-6Al-4V parts exhibited a longer fatigue life than the EBM parts. The difference in the fatigue life behavior was attributed to the presence of the rough surface features that acted as fatigue crack initiation sites in the EBM material [58]. The same material was tested using SLM technology. The fatigue life was significantly lower compared to similar specimens manufactured with the same wrought material. This reduction in the fatigue performance was attributed to a variety of issues, such as the microstructure, porosity, surface finish, and residual stress. Also, a high degree of anisotropy in the fatigue performance was found and was associated with the specimen build orientation [26]. Different SLM stainless steel parts were tested under fatigue regimes. Depending on the material and the post-treatment, the resulting lifetimes were different [60]. Fatigue tests were also performed on the parts manufactured with Stratsys^®^ Polyjet technology using a printed elastomer material. The findings showed the relationship between elongation and expected fatigue life, and that the better surface finish that this technology delivers, contributed to improving the fatigue life of the components [61].

The fatigue life of polylactic acid (PLA) was also investigated, as it is becoming a commonly used thermoplastic in open-source FFF machines for various engineering applications. The samples manufactured in three different orientations were tested. The results showed that the 45° build orientation parts showed a higher fatigue life than the parts built along the X and Y axis [22]. A DOE using different building parameters was used to determine their optimal values on the fatigue performance of the PLA FFF manufactured specimens. It was found that the infill density was the most important parameter, followed by the nozzle diameter and the layer height. Two different infill patterns were compared, with the honeycomb pattern being the best one. The fracture examination evidenced the necessity of post processing the outer layers to maximize the lifespan of the PLA parts [41]. The infill orientation of the FFF ABS parts was investigated by Zieman et al. [52]. The spciments built with the ±45° strategy had the longest fatigue life, followed by the 0, 45, and 90° orientations. The difference between the average cycles to failure was statistically significant for all of the infill orientations at each stress level. The failure modes are similar to those observed in the static tension tests.

During the last years, researchers have tackled the time dependence of the mechanical properties of parts manufactured through AM, specifically their fatigue behavior. Lee and Huang [62] studied the fatigue behavior for different part build orientations of two different materials, ABS and ABS plus. They analyzed the total strain energy absorbed by the specimens, but only one piece at each stress level was tested. Ziemian et al. [63] also published their results regarding the fatigue behavior of fused deposition modelling (FDM) ABS pieces. A fatigue damage analysis and an empirical model of an effective elastic modulus were presented. Senatov et al. [64] published a low cycle fatigue test for the PLA porous scaffolds for bone implants manufactured by FDM, functioning under cyclic loading. The Ultem FDM specimens for several build orientations were investigated by Fischer and Schöppner [65]. Puigoriol-Forcada et al. [66] recently published a study about the flexural fatigue properties of polycarbonate FDM parts. 

To carry out fatigue studies, tests of different types can be selected, where parts of the different configurations are also used. Some examples are those used in the papers previously referred to as bending fatigue tests [66] and tensile fatigue tests [63,21]. In this article, a rotating flexural fatigue test was carried out. The detected lack of references about the influence of other parameters on the fatigue life, and a comprehensive study about the fatigue behavior of the FFF ABS parts has motivated the realization of this study. The innovative approach of this paper lays on the fact that ABS is an almost unexplored material for FFF in terms of fatigue, and the study is performed including a high number of factors in the experimental procedure. The results of the study shall deliver a recommended parameter set in order to maximize the service life of the ABS FFF parts. Furthermore, the influence of the maximum stress characterizing that load shall be studied by constructing the Wöhler curves for the defined optimal parameter set.

## 2. Materials and Methods

The experimental procedure is divided into four parts. First, the experimental factors will be chosen to perform a design of experiments (DOE), so a statistical analysis of the results can be performed. Then, the specimens will be designed and manufactured according to the related experimental matrix. Afterwards, they will be tested and the results will be statistically analyzed. Finally, 24 specimens will be manufactured using the optimal parameters found previously, to represent the Wöhler curve, also known as the S–N fatigue diagram.

## 3. Experimental Factors and Design of Experiments

The fixed manufacturing parameters’ values were selected following different criteria, as shown below:

- Printing temperature. It is the target temperature at which the extruder must operate, in order to have a proper extrusion and to guarantee cohesion in the workpiece. It has been selected according to the manufacturer’s datasheet recommendations.
- Platform temperature. The printing bed must keep this temperature during the whole extrusion process, to improve the quality of the printed pieces by preventing the warping caused by thermal stresses. It was selected according to the manufacturer’s datasheet recommendation.
- Infill angle. It defines the direction of the trajectory that the nozzle follows to fill the internal section limited by the perimeter of the piece. We considered an infill angle of 45°, because it proved to be the best orientation in previous studies [41,67].
- Solid shell. It defines the number of contours present in every layer of the workpieces. The higher the number of layers in the solid shell, the higher the stiffness of the obtained part. The number of solid layer shells were selected so that the specimens had the smallest number of contours, so that it would affect at its minimum the results of the experimental campaign (the influence of the number of contours was not a target parameter in this study).

As for the parameters included in the DOE, they were selected by taking into account both the manufacturer datasheet and the previous investigations concerning the mechanical properties in terms of the fatigue life of other AM parts [21,41,52,67]. They are described below:

- Layer height. It determines the thickness of the layers. Thinner layer heights increase the part quality, leading to a smoother surface but a higher building time. Thicker layers have the opposite effect.
- Nozzle diameter. It determines the diameter of the extruded plastic. This parameter affects the mechanical performance, surface roughness, and cost of the manufactured parts.
- Infill density. It defines the amount of plastic used on the interior part of the print. A higher infill density means more plastic inside the part, leading to a stronger object. This parameter also affects the building time.
- Printing speed. It determines at which speed the print head and the platform move while printing. This setting also determines how fast the filament must be extruded in order to obtain the desired extruded filament width. A higher print speed will lead to a shorter print time.

These variable parameters have been selected by considering both the manufacturer datasheet and the previous investigations concerning the mechanical properties in terms of the fatigue life of other AM parts [21,41,52,67]. The selected fabrication parameters, as well as each of their levels, are shown in Table 1.

A full factorial DOE involving four factors at three levels would consist of 81 experiments (3^4^). The Taguchi method reduces the number of experimental tests and still allows for a statistical analysis of the process parameters and their interactions. Taguchi proposes an experimental plan, in terms of an orthogonal array, giving a certain combination of parameters for each experiment [34,41,45,53].

In this study, the influence of the four factors and the interaction between three of them are studied (A × B, B × C, and A × C). This combination leads to 16 degrees of freedom, therefore the most appropriate orthogonal array is L_27_. The assignment of factors and interactions into the orthogonal matrix was performed using the linear graph for the L_27_ orthogonal array in order to avoid confusion between factors. The assignment was performed as follows: Columns 1, 2, and 5 have been assigned to factors A, B, and C, respectively (according Table 1). Factor D is assigned to column 9. This configuration also allows the parameters A, B, and C to be set in a full factorial DOE, which allows for a detailed study on its influence. The final column assignation is shown in Table 2.

**Figure 1:**
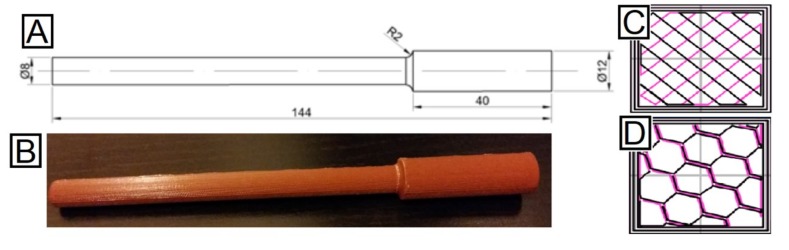
(**A**) Specimens used for the fatigue tests. (**B**) Overview of five specimens manufactured, all of them sharing the same manufacturing parameters. (**C**) Rectilinear infill pattern. (**D**) Honeycomb infill pattern (adapted from [41], with permission from Elsevier).

Additionally, two different infill patterns were introduced in the case study in order to explore their effects on the mechanical behavior (Figure 1C,D). This factor determines the pattern taken by the extruder to deposit the material inside the part, which could be beneficial in some cases [57]. Rectilinear and honeycomb patterns were used, as the results can be compared to those obtained by Gomez-Gras et al. from a similar experimental study performed with PLA specimens, in the same conditions and using the same machine [41,67].

## 4. Test Samples Design and Manufacture

The test specimens were manufactured using a 2.85 mm ABS filament. There is no a specific standard focusing on fatigue testing for additive manufactured plastic parts. Therefore, special specimens have been designed, adapting their dimensions to the possibilities offered by the testing machine (Figure 1A,B). However, the design of the specimens is according to the ASTM D7774 standard [68], which regulates the test method for flexural fatigue properties of plastics.

The test samples were designed using SolidWorks^®^, then sliced using Slic3r, where the different building parameters were set according to the DOE. Finally, the parts were manufactured with a Pyramid dual extruder M^®^ FFF machine (Oxfordshire, UK) oriented along the X axis. A total of 162 samples were manufactured—three repetitions for the 27 parameter set for the two infill patterns. 

## 5. Fatigue Testing

**Figure 2:**
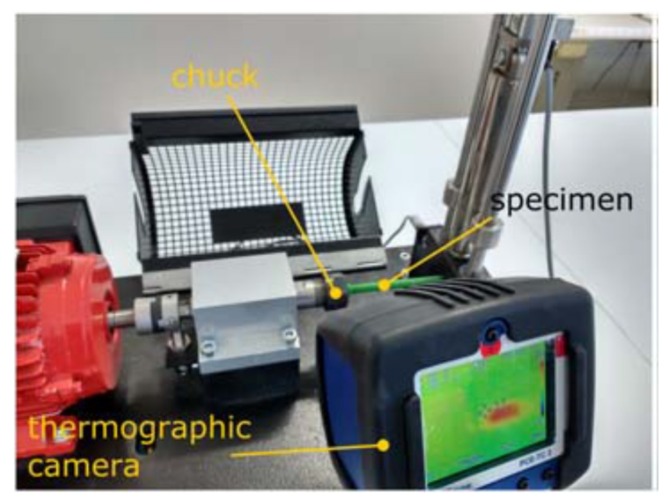
Experimental station (adapted from [41], with permission from Elsevier).

The parts were tested using a GUNT WP 140 machine (Hamburg, Germany) (Figure 2), applying a rotational movement of 2800 min^−1^ and a force of 8 N. The load, applied in the direction perpendicular to the axis of rotation and along the longitudinal axis of the parts, generated a sinusoidal load in the fibers of the specimen. The geometry of the specimens causes failure in the critical section next to the diameter change, where the highest bending moment is being exerted. 

A PCE-TC 3 thermographic camera (Palm Beach, FL, USA) was also installed to observe the changes in temperature of the specimen at the stress concentrator area. Its sensitivity is 0.15 °C and precision is of ±2 °C. Both values are considered admissible for this kind of study, where the temperature can be considered as secondary to characterize the process.

## 6. Statistical Analysis

To determinate the most influential factors in a DOE according to Taguchi’s method, the signal-to-noise (S/N) ratio is used. Signal refers to the target magnitude (number of cycles) and noise represents the variability of that response. As the objective of the experimental plan was to find the parameters that maximize the number of cycles before failure, the aim of the statistical analysis is to maximize the signal and to minimize the noise, thus optimizing the S/N ratio. The ratio was calculated for each experiment using Equation (1), where *η* is the average S/N ratio, *n* is the number of experiments conducted at level *i*, and *y_i_* is the measured value of the property.

(1) $$\eta =-10\cdot \log\left(\frac{1}{n}\overset{n}{\underset{i=1}{\sum }}\frac{1}{y_{i}^{2}}\right)$$

The optimization of the S/N ratio also defines the optimal factors by confirming whether there is a linear correlation between the signal and the S/N ratio, and the standard deviation and the S/N ratio. 

To obtain the influence of each parameter and the interactions in the fatigue life, an analysis of variance (ANOVA) was performed on each parameter using the signal and the noise values. The parameters whose statistical influence was below 10% were not considered. The effect of the levels for each parameter and the interaction on the signal and noise were studied in order to find their influence on the response. The statistical result analysis shall deliver the printing parameters that lead to the highest fatigue lifespan for both of the infill patterns.

### Wöhlers Curve

The optimal parameters found were used to manufacture a whole new set of parts that would be tested to different oscillating bending stress, so that a low-cycle fatigue study can be performed. The obtained results would lead to the determination of a Wöhler curve of the parameters set.

## 7. Results

In this section, the results obtained are presented in four subsections. First, the fatigue results acquired using Taguchi’s DOE, and a fractography study are presented. Then, the comparison between the two infill patterns is shown, and finally, the resulting Wöhler curve is discussed. 

### 7.1. Fatigue Results

The signal and noise response for each experiment are shown in Table 2. There was no correlation between the signal and the S/N ratio, or the noise and the S/N ratio. Therefore, a dual response approach was needed, so the factors that maximize the signal response and minimize the noise can be determined.

The results showed that the most influential factor in the signal was the infill density, for both of the infill patterns (42.2% for rectilinear and 72.4% for honeycomb), as it happened in the previous study done for PLA material [41]. The interaction between the layer height and the nozzle diameter was the next most influential in the number of cycles (18.8% for rectilinear and 17.3% for honeycomb). The other factors and interactions were declared non-influential, due to the fact that their influence was lower than 10%.

**Figure 3:**
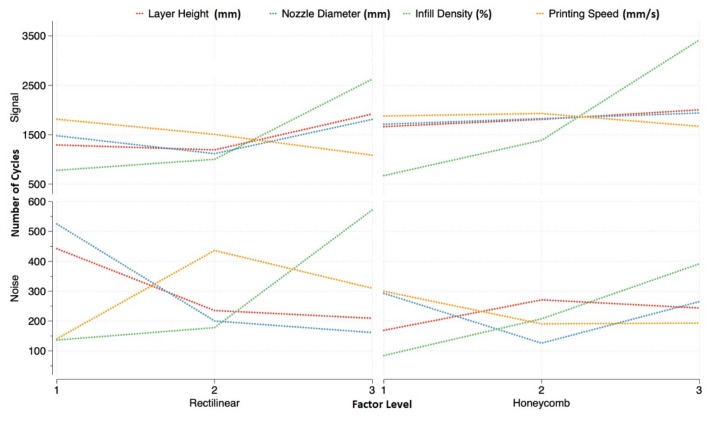
Factor effect on signal and noise for both infill patterns.

The noise results exhibited the same trend, with the infill density being the most influential factor (25.3% for rectilinear and 40% for honeycomb), followed by the interaction between the layer height and the nozzle diameter (20.0% for rectilinear and 15.8% for honeycomb). However, the nozzle diameter also exhibited a significant effect in both infills (17.4% for rectilinear and 13.4% for honeycomb). Again, the factors and interactions with an influence lower than 10% were ignored. The effect for each factor according to their level can be observed in Figure 3, where the evolution of all of the results are joined by discontinuous lines to guide the eye of the reader.

### 7.2. Optimal Factors for Rectilinear Infill Pattern

The results showed that the highest lifespan, using the rectilinear infill pattern, was obtained when layer height, nozzle diameter, and infill density were at their highest level. On the other hand, the lowest variance was obtained when the infill density was at the lowest level, and the layer height and nozzle diameter at their mid or highest level, due to the lower difference shown. 

**Figure 4:**
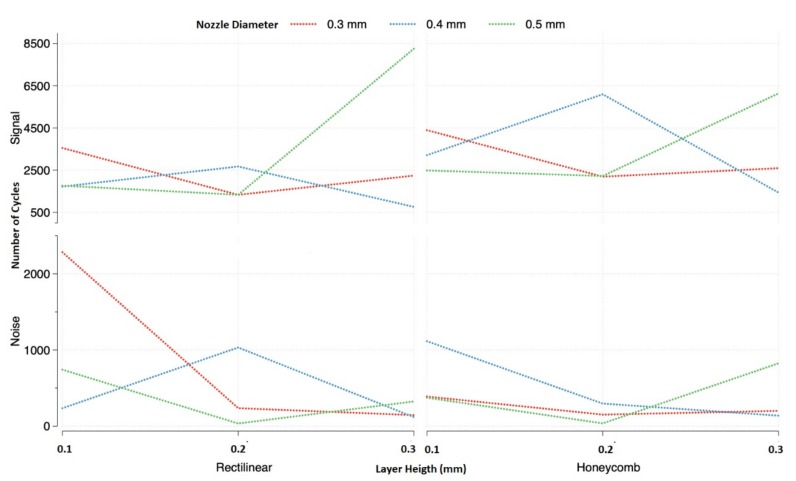
Interaction plots between nozzle diameter and layer height. Effect on signal and noise using rectilinear infill pattern on the left and honeycomb infill pattern on the right both at their highest level (75%).

The interaction between the layer height and the nozzle diameter proved to be significant in the signal and the noise of the response. Since the significance of the infill density factor on the signal is higher than on the noise, its optimal level can be defined at 75%—level 3 (Figure 4).

It could be observed that the interaction between the layer height and nozzle diameter was complex, as the effects on the signal and the noise could not be separated from one another. The maximum signal is obtained when the nozzle diameter and layer height are selected at their highest level, observed in Figure 4. On the other hand, the effect of the layer height on the noise was minimized when the nozzle diameter was at its highest level, and its influence was almost as important as the interaction. Therefore, to minimize the variability of the signal, the nozzle diameter must be at its highest level.

The experiences that presented the best configurations of parameters for the rectilinear pattern were 9, 18, and 27 (Table 2). What those configurations have in common is that the nozzle diameter and the infill density are at their highest levels. It was observed that the best result for the rectilinear infill was experiment number 27, which corresponded to the three most influential factors at their highest level. This set of parameters presented an average life of 8262 cycles with just a 3.9% variance, which made this configuration the best one.

### 7.3. Optimal Factors for Honeycomb Infill Pattern

A similar analysis was performed for the honeycomb pattern results. The nozzle diameter and layer height maximized the lifespan at their middle and highest level and, like the rectilinear pattern, the infill density at its highest. The lowest noise was observed when the layer height was at its middle or highest level, nozzle diameter at its middle level, and infill density at its lowest (Figure 3). 

The same situation using honeycomb happened as with a rectilinear pattern. The importance of the infill density in the signal was higher than in the noise, so, in order to maximize the cycles to failure, the infill pattern should be the highest—75% (Figure 4 right). 

The same interaction was found to be significant using honeycomb, but in this case, there was no level for any of the factors that minimized the effect of the other. In order to maximize the signal, the nozzle diameter and the layer height needed to be at the same level. Minimizing the variance of the signal appeared to be more complicated; depending on the value of the nozzle diameter, the layer height could be at any of its levels. The lowest noise was found when the layer height was at its highest level and the nozzle diameter at its middle one, and vice versa. The best combinations of factors that would magnify the signal and minimize the noise should be when both factors are at levels 2 and 3.

The experiences that presented these combinations were 15, 18, 24, and 27. From these four, numbers 15 and 27 presented the highest life cycles, which were almost identical (Table 2). But experience 15 presented the lowest variance of the two (4.9% for experience 15 and 13.4% for experience 27). This difference in variance made the 15th experience the optimal one.

### 7.4. Fractography

**Figure 5:**
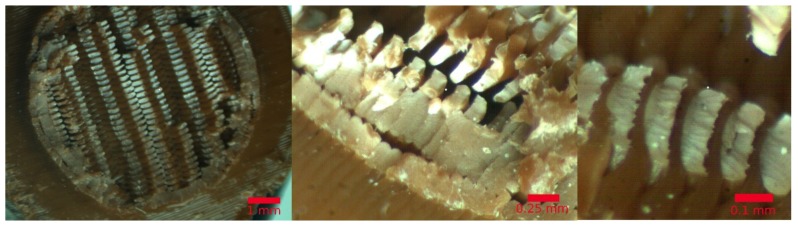
Image of the fractured area of the specimen.

Photographs of the broken specimens were taken after the fatigue tests. They were taken with a MOTIC SMC (Hong Kong, China) binocular loupe equipped with a MOTICAM 3 digital camera. The photographs showed singular aspects that describe the breaking mode found in the specimens tested. In all of the cases, the crack began around the area near the first or last printed layer, observed in Figure 5, on the left. This implied that the extruded filaments that were forming the curvature of the specimens acted as stress concentrators, so the cracks were formed there and then propagated inside the part.

In all of the cases, the type of break observed was ductile on the entire XY plane. The details of the fatigue marks are easily observed in Figure 5 in the middle and left, where the photographs from different specimens using different printing parameters are presented. However, it can be assessed that the type of break was the same in all of them. The propagation of the cracks as a combination of the bending and shear stress defined the failure mode of this type of material, as has already been discussed by other authors [64].

### 7.5. Infill Pattern Comparison

**Figure 6:**
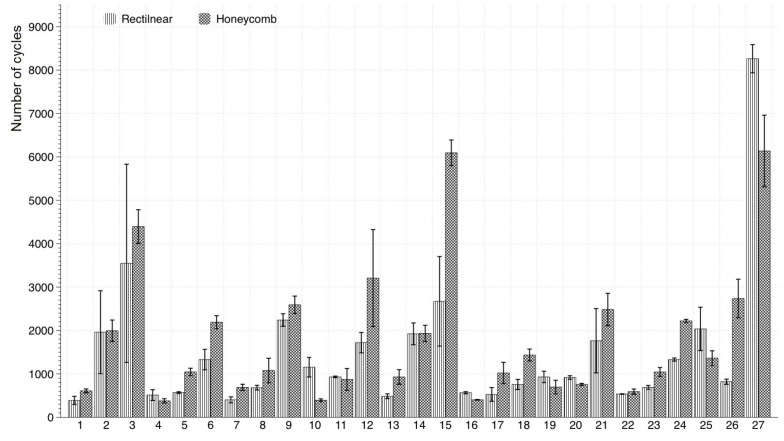
Lifespan comparison chart between rectilinear and honeycomb infill pattern.

Figure 6 shows the comparison between the two infill patterns for all of the experiments. It can be observed that, depending on the factor levels, the difference in life cycles was significant and no relation was noticeable. However, the honeycomb configuration shows a better lifespan than the rectilinear in almost all of the configurations. Test number 27 showed the maximum life for both infill architectures, but the lifespan using the rectilinear pattern was 25% higher than using the honeycomb pattern.

On the other hand, there were two experiences that showed the highest lifespans using honeycomb pattern, numbers 15 and 27. Also, configuration number 15 showed that using the honeycomb pattern resulted in an over 50% of lifespan in comparison with the rectilinear configuration. 

### 7.6. Wöhler Curve

The analyzed results led to the conclusion that there was an optimal combination of parameters in the defined DOE, summarized in Table 3. This set of conditions was applied to print a second set of specimens, which were tested to different levels of bending stress, obtained by applying different forces at the specimen extreme point. Table 4 shows the eight different levels of force and the maximum bending stress to which the specimen were subjected in the stress concentrator area, calculated considering that the specimen can be modelled as a cylindrical cantilever.

With this data, different fatigue tests to construct the Wöhler curve were carried out at each of the indicated stress levels [38]. Following the protocol established by Wirsching, M.C. [69], and also that applied in our previous study [41], five repetitions were performed for each stress level, except for 28.7 MPa, as this stress was already tested for the results of the DOE analysis. 

The least-square regression model was used to fit the linearized version of the potential Wöhler curve (Equation (2))

(2) $$\log\left(2N_{f}\right)=-\frac{1}{b}\log\left(S_{f}\right)+\frac{1}{b}\log\left(S_{a}\right)$$

where $\log\left(S_{a}\right)$ is the independent variable, $\log\left(2N_{f}\right)$ is the dependent variable, the slope is $\frac{1}{b}$, and interception point is $\frac{-1}{b}\log\left(S_{f}\right)$. Thus, the S–N curve equation is Equation (3).

(3) $$S_{a}=S_{f}{\left(2N_{f}\right)}^{b}$$

**Figure 7:**
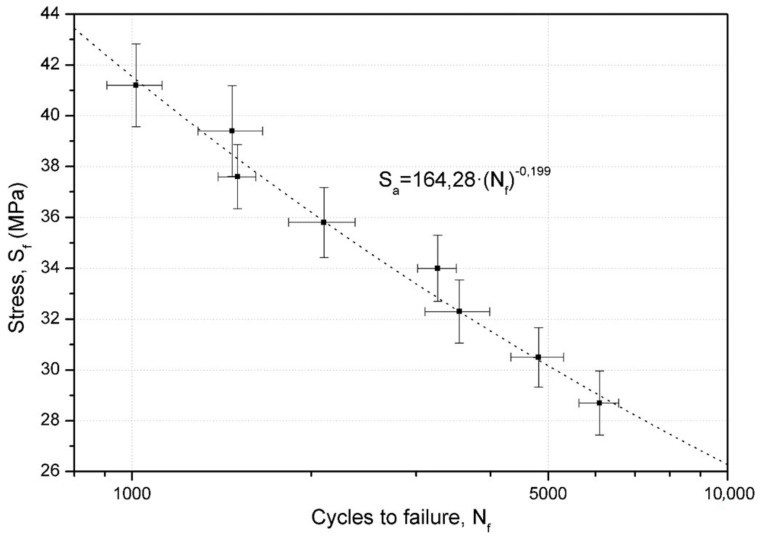
Wöhler curve for specimens manufactured with honeycomb infill, 75% infill density, 0.4 mm diameter nozzle, and 0.2 mm layer height.

A potential curve, corresponding to Equation (3), was deduced from the testing, with a R^2^ = 0.9814, and is represented in Figure 7. Furthermore, the model used in this figure is only valid for the low cycle fatigue domain.

## 8. Discussion

The results obtained showed that the infill density is the most important parameter affecting the live span of the ABS FFF produced parts. The other parameters studied do not have that much impact on the cycles to fail on their own, but instead on their interactions. It is also important that the influence of factors and interactions, for signal and noise, are the same and in the same order for the two infill patterns.

It is evident that when parts are more uniform or continuous, as the injected ones, their mechanical properties are better. Voids are always present when manufacturing parts using FFF technology, even if the parts are manufactured completely as solid. So comprehensively, the infill density has been found to be the most important factor affecting the life of a part—the more density, the more continuous, and the more life cycles the part can stand.

The interaction between the layer height and nozzle diameter has been found to be important. These two parameters also affect the continuity of the part. The higher they are, the more continuous the part is, as there are fewer interfaces inside the part. The nozzle diameter makes the extruded filament bigger, so the part is more continuous with lesser voids inside. Bigger layer heights cause the part to be manufactured with fewer layers, which is also more continuous. This result was also observed on the PLA specimens [41].

The printing speed does not affect the fatigue performance of the ABS FFF manufactured parts. This conclusion is reasonable, as the speed values that are tested in the experimental plan are not significantly different.

The difference between the two infill parameters in the cycles to failure is not evident. Overall, the honeycomb specimens are proven to have better results. However, this result varies according to the other parameters. For instance, if experiment number 27 is compared, the rectilinear pattern shows better results. This may be caused by the fact that the stress created by the load during the experiment is aligned with the layers, as specimens are printed along the X axis. The rectilinear pattern positions extruded filaments at 45° along the load, which causes an equal distribution of the stress along the plane, so the part is stronger. On the other hand, the honeycomb pattern does not transmit the stress the same way, or, at least, it is not proportional along the plane, making this pattern weaker in this case.

The evolution of the fatigue life versus stress amplitude of the selected printing conditions could be properly described by Wöhler’s potential equation, as was also found in PLA [67]. This means that the selected range of the stress amplitudes corresponds to the same fatigue regime, elastic fatigue in this case, and no fatigue limit was observed.

## 9. Conclusions

In the present paper, the fatigue life cycles of the ABS parts manufactured with FFF technology using different building parameter configurations have been analyzed. Test samples have been built varying in layer height, nozzle diameter, infill density, printing speed, and infill pattern. The results obtained have confirmed the following:

1. The fatigue performance depends on the building parameters. This means that, by controlling the building parameters, the mechanical behavior of the FFF parts can also be controlled.
2. The infill density is the most important factor for the two infills structures studied. The fatigue life increases as the infill does. The infill strengthens the part causing an increased life. For any combination of building parameters, the higher the density inside the part, the higher the life span.
3. Selecting the right building parameters is not an easy task; as proven in this study, the selection of the right value of different parameters can increase the mechanical properties considerably, but some generalization can be extracted.
4. The improvement of the life of FFF parts is achieved when the parts are manufactured to be as continuous as possible, and also, when the direction of the extruded filaments or the infill pattern inside the part make the tension distribute equally.
5. This paper has also presented the S–N curve associated with the best 3D printing parameters. This curve can be adjusted by a simple Wöhler model, meaning that, at the tested stress levels, the ABS specimens are working inside the elastic region.
6. Further studies are needed to understand how the parameters studied, and others, affect the fatigue performance of FFF ABS produced parts. However, the obtained results in this study (and others with different materials) are expected to be similar for other FFF thermoplastics, not in value, but how the factors affect the life cycle.

## 10. Data Availability

The raw/processed data required to reproduce these findings cannot be shared at this time, as the data also forms part of an ongoing study.

## Figures and Tables

**Table 1:** Fabrication factors considering levels for experimentation.

Fixed Manufacturing Factors			Variable Manufacturing Factors					
Factor	Value	Unit	Factor	Symbol	Level			Unit
					1	2	3	
Printing temperature	230	°C	Layer height	A	0.1	0.2	0.3	mm
Platform temperature	100	°C	Nozzle diameter	B	0.3	0.4	0.5	mm
Infill angle	45	°	Infill density	C	25	50	75	%
Number of perimeters	2	-	Printing speed	D	25	30	35	mm/s
Solid layers shell	3	-	Fill Pattern	E	Rectilinear	-	Honeycomb	-

**Table 2:** L_27_ matrix column assignation along with signal and noise values for the life cycles of rectilinear and honeycomb infill patterns.

Test #	Factor				Rectilinear		Honeycomb	
	Layer Height (mm)	Nozzle Diameter (mm)	Infill Density (%)	Printing Speed (mm/s)	Signal (Num. of Cycles)	Noise (Num. of Cycles)	Signal (Num. of Cycles)	Noise (Num. of Cycles)
1	0.1	0.3	25	25	388	94	609	45
2	0.1	0.3	50	30	1961	955	1995	246
3	0.1	0.3	75	35	3549	2284	4395	389
4	0.1	0.4	25	30	512	124	378	50
5	0.1	0.4	50	35	569	20	1045	85
6	0.1	0.4	75	25	1330	236	2191	151
7	0.1	0.5	25	35	401	69	689	72
8	0.1	0.5	50	25	683	54	1078	283
9	0.1	0.5	75	30	2241	144	2592	201
10	0.2	0.3	25	30	1154	225	393	32
11	0.2	0.3	50	35	931	18	872	251
12	0.2	0.3	75	25	1720	235	3208	1116
13	0.2	0.4	25	35	484	55	929	168
14	0.2	0.4	50	25	1923	251	1933	187
15	0.2	0.4	75	30	2672	1033	6095	296
16	0.2	0.5	25	25	566	25	402	8
17	0.2	0.5	50	30	527	158	1021	245
18	0.2	0.5	75	35	756	117	1435	137
19	0.3	0.3	25	35	930	131	696	157
20	0.3	0.3	50	25	916	41	757	25
21	0.3	0.3	75	30	1764	741	2484	373
22	0.3	0.4	25	25	536	6	591	60
23	0.3	0.4	50	30	689	44	1044	102
24	0.3	0.4	75	35	1330	35	2222	36
25	0.3	0.5	25	30	2037	500	1362	170
26	0.3	0.5	50	35	819	60	2737	445
27	0.3	0.5	75	25	8262	324	6137	825

**Table 3:** Optimal combination of factors and levels to maximize the expected cycles to failure.

Parameter	Value
Infill pattern	Honeycomb
Fill density	75%
Nozzle diameter	0.4 mm
Layer height	0.2 mm

**Table 4:** Forces applied for the Wöhler curve tests and maximum stress levels.

*F* (N)	M_max_ (N-mm)	*σ*_max_ (MPa)
8.0	832	28.7
8.5	884	30.5
9.0	936	32.3
9.5	988	34.0
10.0	1040	35.8
10.5	1092	37.6
11.0	1144	39.4
11.5	1196	41.2
